# Cervical and Breast Cancer Screening Among Mexican Migrant Women, 2013

**DOI:** 10.5888/pcd13.160036

**Published:** 2016-08-11

**Authors:** Natalie Guerrero, Xiao Zhang, Gudelia Rangel, J. Eduardo Gonzalez-Fagoaga, Ana Martinez-Donate

**Affiliations:** Author Affiliations: Natalie Guerrero, Xiao Zhang, School of Medicine and Public Health, University of Wisconsin–Madison, Madison, Wisconsin; Gudelia Rangel, J. Eduardo Gonzalez-Fagoaga, Mexico Section, U.S.–Mexico Border Health Commission, Tijuana, Mexico.

## Abstract

**Introduction:**

Information on cervical and breast cancer screening among Latinas in the United States is limited. Even less information is available on screening practices of migrant women who engage in circular migration. We examined rates of cervical and breast cancer screening and the extent to which sociodemographics and other characteristics explain screening practices of Mexican migrant women who return to Mexico from the United States.

**Methods:**

We used data from a cross-sectional probability survey of Mexico-born migrant women who returned, through Tijuana, to Mexico from the United States in 2013. The sample consisted of women who returned involuntarily (via deportation) or voluntarily; 177 reported authorized documentation status, and 36 reported unauthorized documentation status in the previous 12 months. Descriptive statistics were calculated and logistic regressions were estimated.

**Results:**

Of 36 undocumented migrant women, 8 (22.2%) had a Papanicolaou test and 11 (30.6%) had a mammogram in the previous year; of 177 documented migrants, 83 (46.9%) had a Papanicolaou test and 68 (38.4%) had a mammogram. Undocumented migrants were less likely than documented migrants to receive a Papanicolaou test (odds ratio [OR] = 0.29; 95% confidence interval [CI], 0.12–0.67); the likelihood was similar after adjustment for sociodemographic, migration, and acculturation factors (adjusted OR = 0.33; 95% CI, 0.12–0.90). Having health insurance (adjusted OR = 4.17; 95% CI, 1.80–9.65) and a regular source of health care (adjusted OR = 2.83; 95% CI, 1.05–7.65) were significant predictors of receiving a mammogram but not a Papanicolaou test.

**Conclusion:**

Public health programs are needed to improve access to cervical and breast cancer screenings for Latina migrant women in general and undocumented circular migrants in particular.

## Introduction

Foreign-born Latinas are more likely than US-born Latinas and white women to receive a diagnosis of late-stage cervical or breast cancer (1,2), probably because cancer screenings are underused by this population. Latinas not born in the United States have lower rates of cancer screening than US-born Latinas, white women, and black women (3–5). Undocumented Latinos in the United States also underuse cancer screening services (6,7). This underuse of screening services places undocumented Latina immigrants at greater risk of late-stage cancer diagnoses compared with their documented counterparts. In addition, mobility may be a barrier to accessing health services (8). One-third of Mexican migrants engage in circular migration (9), defined as repeated migrations between point of origin and destination. Yet, to our knowledge, no research has examined health care use by Mexican women who engage in circular migration.

The US Preventative Services Task Force (USPSTF) recommends a mammogram every 2 years for women aged 50 to 74 and a Papanicolaou (Pap) test every 3 years for women aged 21 to 65 years. To address the need for additional research on rates of, and factors associated with, receipt of cancer screening services by Mexican migrant women, we examined differences in receipt of cervical and breast cancer screening by documented and undocumented Mexican circular migrants. First, we compared the prevalence of self-reported, previous 12-month receipt of cervical and breast cancer screening between documented and undocumented Mexico-born Latinas who returned to Mexico from the United States. Second, we examined the extent to which sociodemographic and other characteristics explain screening practices, with an emphasis on documentation status. Because research on migrants shows that documentation status, health insurance, regular source of care, and acculturation are associated with use of preventive health care services (10–13), we hypothesized that these factors would also predict the level of use of cervical and breast cancer screening.

## Methods

### Sample

We used 2013 data from the project *Migrante*, which comprised a series of cross-sectional probability surveys of Mexican migrants in Tijuana from 2007 to 2015 (http://migrante.weebly.com). One-quarter of migrants who travel south from the United States to Mexico travel through Tijuana (14). The *Migrante* surveys used a multistage sampling design, and samples consisted of migrants surveyed at key transit points in Tijuana. Eligible respondents were aged 18 years or older, were born in Mexico or another Latin American country, were fluent in Spanish, and had never before participated in the *Migrante* survey. Mexican migrants were approached consecutively as they were crossing through the sampling point and then screened for eligibility. Details on survey methods are described elsewhere (9). The Health Sciences Minimal Risk Institutional Review Board at the University of Wisconsin-Madison and the institutional review board of the Mexico Section of the US–Mexico Border Health Commission approved the project.

The *Migrante* survey conducted in 2013 focused on the use of health care services. In that year, 4,215 eligible male and female migrants were screened for eligibility (Figure) and 2,441 agreed to participate (58% response rate). For this study, we analyzed data from women migrants returning from the United States. These data came from 2 groups of women in 2 migration flows: one group comprised Mexico-born Latinas returning to Mexico from the United States via deportation (deported flow), and the other group comprised Mexico-born Latinas recently arrived from the United States on their way to their communities of origin in Mexico (southbound flow). The deported-flow migrants were recruited for the study in Tijuana’s deportation station; this group consisted of 61 migrants who were intercepted during their attempt to cross the border into the United States or who crossed successfully but were later deported. Most of the 191 southbound-flow migrants recruited for this study were returning voluntarily; they were permanently or temporarily established in the United States, and they were heading to their communities of origin in Mexico. Ten women in the southbound-flow, however, were returning to Mexico because of deportation.

**Figure:**
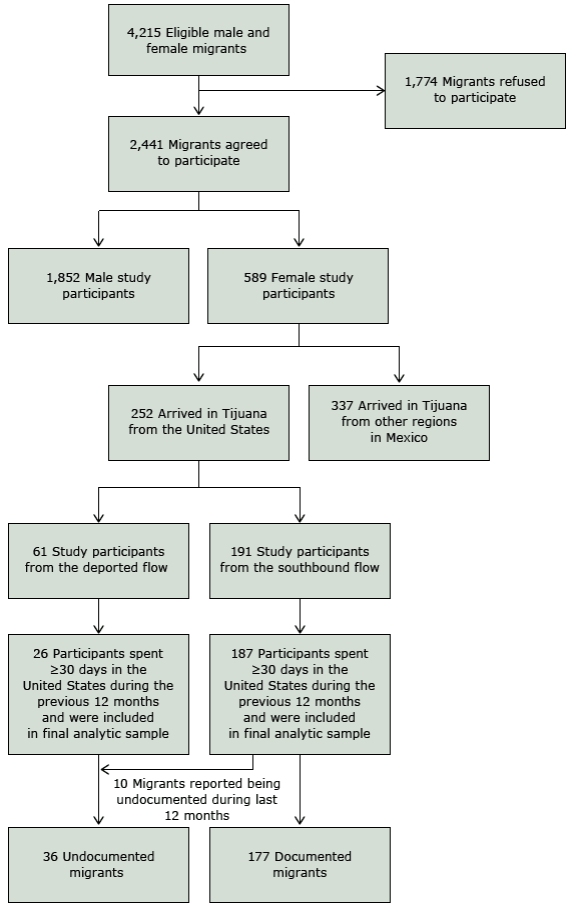
Participant recruitment for study on cervical and breast cancer screening among Mexican migrant women, 2013. Data were collected through the project *Migrante*, which comprised a series of cross-sectional probability surveys of Mexican migrants in Tijuana from 2007 to 2015 (http://migrante.weebly.com).

By design, our sample consisted of circular migrants, defined as migrants who had completed a migration cycle; that is, they left their communities of origin, moved to the United States, and returned to Mexico either voluntarily or involuntarily (because of deportation). Immigrants (those who travel in only one direction [south to Mexico] and never return to the United States) were not included in *Migrante* sampling frameworks for southbound-flow or deported-flow migrants. Therefore, we use the term “circular migrants” throughout this article.

We restricted the analytical sample to migrants who had spent 30 days or more in the United States during the previous 12 months. We imposed this restriction because most migrants spend 30 days or more in this country; the restriction should increase the relevance of our findings to health policies in the United States. Another reason for this restriction was that data on some predictors of health care access were available for this subset. After imposing this restriction, data on 39 respondents were excluded from the analysis (35 from the deported flow; 4 from the southbound flow). The final analytical sample consisted of 36 undocumented women (10 from southbound flow; 26 from the deported flow) and 177 documented women (all from the southbound flow).

### Measures

The primary outcome of interest was self-reported receipt of mammogram and Pap test in the previous 12 months. Survey participants reported whether they had received these services in the previous 12 months, and if yes, in which country or countries. The main predictor was self-reported documentation status in the United States in the previous 12 months. Undocumented migrants were defined as all women from the deported group and women from the southbound group who answered yes to the question “During the last 12 months in the United States, were you undocumented any of the time?”

We collected self-reported data on the following sociodemographic characteristics: age, marital status, and education. We analyzed data on mammograms in two ways: overall and by age group (younger than 50 and 50 or older). We also analyzed data on Pap tests overall and by age group (younger than 21 and 21 or older). Marital status was categorized into unmarried, married but not living with a spouse in the United States, and married and living with a spouse in the United States. Participants reported their highest level of education completed, and a binary variable was created to stratify those who had completed high school and those who had not. We also collected data on migration characteristics, health care characteristics, and level of acculturation. Participants reported the amount of time spent in the United States in the previous 12 months (recoded into 30 d to <6 mo, 6 mo to <12 mo, or 12 mo) and in their lifetime (recoded into <1 y, 1–4 y, 5–9 y, and ≥10 y). Survey participants reported having or not having a regular source of health care and any form of health insurance in the previous 12 months in the United States. Acculturation was measured by using a scale adapted from Finch et al (15). A continuous variable was derived from 4 questions that assessed English proficiency (for example, “In the United States, what language did you prefer to speak?”). For each question, participants received a score of 0 (Spanish or a native language always or most of the time), 1 (Spanish or a native language as much as English), or 2 (English always or most of the time). The higher the score (range 0–8), the greater the acculturation to the United States.

### Statistical analysis

First, we compared the characteristics and rates of previous 12-month cervical and breast cancer screening between documented and undocumented women. Descriptive statistics (mean, standard deviation) were determined for continuous variables. Frequency distributions were calculated for categorical variables. To assess differences between the groups, we used *t* tests and χ^2^ tests. Second, we examined the extent to which differences in characteristics explained differences in receipt of Pap test and mammogram between the documented and undocumented women. Multivariate logistic regressions were estimated. A block-by-block approach was used to examine the extent to which sets of variables explained differences in screening receipt between documented and undocumented women. The primary analysis consisted of a series of 4 logistic regression models to test the relationship between documentation status and screening receipt, ranging from an unadjusted to a fully adjusted model. The first model included only a term for documentation status. The next three models sequentially added covariates for demographic variables (age, marital status, education), migration characteristics (time spent in the US in the previous 12 months and during lifetime), health care access (health insurance status, regular source of care), and acculturation. Documented status was used as the reference category, and other reference categories included unmarried marital status, living in the United States for less than 30 days in the previous year, and living in the United States for less than 1 year during a lifetime. Analyses were completed with the entire analytical sample. Sensitivity analyses were performed with subsamples formed according to the latest screening recommendations (ie, aged ≥21 for Pap test and aged ≥50 for a mammogram) to examine the robustness of the findings. The results did not change substantially, but statistical power was significantly reduced. For that reason, we focused our study on the entire analytical sample. Analyses were performed with STATA/SE version 14.0 (StataCorp LP). Statistical significance was determined at the .05 level.

## Results

Of undocumented migrants, 22.2% (8 of 36) reported having a Pap test in the previous year, compared with 46.9% (83 of 177) of documented migrants, all of whom were aged 21 or older (Table 1). Of undocumented migrants, 30.6% (11 of 36) had a mammogram, and 38.4% (68 of 177) of documented migrants had a mammogram; this difference was not significant. We found no significant differences in mammogram receipt between women younger than 50 and women 50 or older. Of women who received either of these services, most received them only in the United States. Of 91 women who received a Pap test, 65 received it only in the United States, 21 only in Mexico, and 4 in both countries. For mammograms, 79 women received the screening: 61 only in the United States, 14 only in Mexico, and 3 in both countries. One woman received both a mammogram and Pap test but received neither in the United States or Mexico. In general, undocumented migrants were significantly younger and significantly more likely to have been in the United States for the entire previous 12 months.

**Table 1:** Characteristics of Mexican Female Migrant Study Participants, by Documentation Status, Tijuana, Mexico, 2013^a^^, ^b^, ^c

Characteristic	Undocumented^d^, n (%) (N = 36)	Documented^e^, n (%) (N = 177)	*P* Value^f^
**Cancer screening during previous 12 months**			
Had a mammogram	11 (30.6)	68 (38.4)	.26
Aged <50 y	8 (22.2)	26 (14.7)	.57
Aged ≥50 y	3 (8.3)	42 (23.7)	.66
Mammogram by location			
No receipt of service	25 (69.4)	99 (55.9)	.62
Only in the United States	9 (25.0)	52 (29.4)	
Only in Mexico	1 (2.8)	13 (7.3)	
In both the United States and Mexico	1 (2.8)	2 (1.1)	
In neither United States nor Mexico	0 (0)	1 (0.6)	
Data missing	0 (0.0)	10 (5.6)	
Had a Papanicolaou test	8 (22.2)	83 (46.9)	.001
Aged <21 y	0 (0)	(0)	NA
Aged ≥21 y	8 (22.2)	83 (46.9)	.001
Papanicolaou test by location			
No receipt of service	28 (77.8)	84 (47.5)	.046
Only in the United States	7 (19.4)	58 (32.8)	
Only in Mexico	1 (2.8)	20 (11.3)	
In both the United States and Mexico	0 (0.0)	4 (2.3)	
In neither United States nor Mexico	0 (0.0)	1 (0.6)	
Data missing	0 (0.0)	10 (5.6)	
**Sociodemographic characteristics**			
Age, mean (SD), y	38.9 (8.1)	47.9 (14.0)	<.001
Marital status			
Unmarried	18 (50.0)	69 (39.0)	.17
Married, not living with spouse	6 (16.7)	16 (9.0)	
Married, living with spouse	12 (33.3)	82 (46.3)	
Data missing	0 (0.0)	10 (5.6)	
Completed high school	9 (25.0)	69 (39.0)	.08
**Migration characteristics**			
Time spent in the United States during previous 12 months			
30 d to <6 mo	3 (8.3)	27 (15.3)	.003
6 mo to <12 mo	10 (27.8)	83 (46.9)	
12 mo	23 (63.9)	57 (32.2)	
Data missing	0 (0.0)	10 (5.6)	
Time spent in the United States during lifetime, y^g^			
<1	0 (0)	10 (5.6)	.07
1–4	0 (0)	16 (9.0)	
5–9	5 (13.9)	18 (10.2)	
≥10	30 (83.3)	107 (60.5)	
Data missing	1 (2.8)	26 (14.7)	
**Health care access^g^**			
Has had any health insurance during previous 12 m	14 (40.0)	95 (53.7)	.06
Has a regular source of health care	26 (74.3)	114 (64.4)	.68
**Acculturation**			
Level of acculturation based on language^h^, mean (SD), y	1.9 (2.4)	1.3 (1.8)	.11

Abbreviations: NA, not applicable; SD, standard deviation.

a Data source: Project *Migrante*, which comprised a series of cross-sectional probability surveys of Mexican migrants in Tijuana from 2007 to 2015 (http://migrante.weebly.com).

b Sample restricted to migrants who had spent ≥30 days in the United States during the previous 12 months.

c Values are number (percentage) unless otherwise indicated.

d Undocumented defined as all women from the deported flow and women from the southbound flow who answered yes to the question “During the last 12 months in the United States, were you undocumented any of the time?” The deported flow comprised Mexico-born Latinas returning to Mexico from the United States via deportation, and the southbound flow comprised Mexico-born Latinas recently arrived from the United States on their way voluntarily (not via deportation) to their communities of origin in Mexico.

e Documented defined as women from the southbound flow who answered no to the question “During the last 12 months in the United States, were you undocumented any of the time?”

f χ^2^ and *t* tests used to determine *P* values.

g One women in the deported flow did not answer this question.

h On a scale of 0 to 8, with 0 = lowest level of acculturation, 8 = highest level of acculturation. Scale adapted from Finch et al (15).

In Model 1 (unadjusted) of the logistic regression analyses for Pap test, undocumented migrants were significantly less likely than documented migrants to receive a Pap test (odds ratio [OR] = 0.29; 95% confidence interval [CI], 0.12–0.67) (Table 2). In Model 2, when age, marital status, and education were included, we found no change from Model 1 (adjusted OR = 0.30; 95% CI, 0.12–0.72). In Model 3, which included variables for time spent in the United States, health insurance status, and regular source of care status, the adjusted OR of documented status was 0.35 (95% CI, 0.13–0.95). In Model 4, which included acculturation level, we found no change from Model 3 (adjusted OR = 0.33; 95% CI 0.12–0.90). Documentation status was the only predictor significantly associated with the odds of Pap test receipt in any of the models.

**Table 2:** Factors Associated With Receipt of Papanicolaou Test Among Mexican Migrant Women, Tijuana, Mexico, 2013^a^^, ^b

Factor	Model 1, AOR (95% CI)	Model 2, AOR (95% CI)	Model 3, AOR (95% CI)	Model 4, AOR (95% CI)
**Main predictor**				
Documented^c^ during previous 12 mo	Reference	Reference	Reference	Reference
Undocumented^d^ during previous 12 mo	0.29 (0.12–0.67)	0.30 (0.12–0.72)	0.35 (0.13–0.95)	0.33 (0.12–0.90)
**Sociodemographic characteristics**				
Age		0.99 (0.97–1.02)	1.00 (0.97–1.03)	1.00 (0.97–1.03)
Marital status				
Unmarried		Reference	Reference	Reference
Married, not living with spouse		1.72 (0.63–4.71)	2.00 (0.65–6.14)	2.16 (0.70–6.69)
Married, living with spouse		1.51 (0.82–2.79)	1.46 (0.72–2.98)	1.56 (0.76–3.22)
Completed high school		1.77 (0.94–3.32)	1.62 (0.79–3.34)	1.46 (0.69–3.07)
**Migration characteristics**				
Time spent in the United States in the previous 12 months				
30 d to <6 mo			Reference	Reference
6 mo to <12 mo			0.50 (0.17–1.52)	0.53 (0.17–1.62)
12 mo			0.57 (0.18–1.81)	0.60 (0.18–1.92)
Time spent in the United States during lifetime, y				
<1			Reference	Reference
1–4			0.73 (0.13–4.23)	0.78 (0.14–4.47)
5–9			0.41 (0.07–2.44)	0.41 (0.07–2.44)
≥10			0.68 (0.13–3.59)	0.59 (0.11–3.16)
**Health care access**				
Had any health insurance during previous 12 mo			1.92 (0.90–4.09)	1.88 (0.88–4.03)
Has a regular source of health care			2.10 (0.88–5.03)	2.19 (0.91–5.28)
**Acculturation**				
Level of acculturation based on language^e^				1.11 (0.91–1.36)

Abbreviations: AOR, adjusted odds ratio; CI, confidence interval.

a Data source: Project *Migrante*, which comprised a series of cross-sectional probability surveys of Mexican migrants in Tijuana from 2007 to 2015 (http://migrante.weebly.com).

b Sample restricted to migrants who had spent ≥30 days in the United States during the previous 12 months.

c Undocumented defined as all women from the deported flow and women from the southbound flow who answered yes to the question “During the last 12 months in the United States, were you undocumented any of the time?” The deported flow comprised Mexico-born Latinas returning to Mexico from the United States via deportation, and the southbound flow comprised Mexico-born Latinas recently arrived from the United States on their way voluntarily (not via deportation) to their communities of origin in Mexico.

d Documented defined as women from the southbound flow who answered no to the question “During the last 12 months in the United States, were you undocumented any of the time?”

e On a scale of 0 to 8, with 0 = lowest level of acculturation, 8 = highest level of acculturation. Scale adapted from Finch et al (15).

In Models 1 through 4 of the logistic regression analyses for mammogram receipt, we found no significant differences between documented migrants and undocumented migrants in the likelihood of receiving a mammogram (Table 3). After adjustment for sociodemographic factors, migration characteristics, and acculturation level (Model 4), age was significantly associated with increased odds of mammogram receipt (adjusted OR = 1.06; 95% CI, 1.02–1.09). Having health insurance (adjusted OR = 4.17; 95% CI, 1.80–9.65) and a regular source of health care (adjusted OR = 2.83; 95% CI, 1.05–7.65) were significant predictors of mammogram receipt. Model 4 also demonstrated significantly increased odds of mammogram receipt with increased level of acculturation (adjusted OR = 1.25; 95% CI, 1.01–1.55).

**Table 3:** Factors Associated With Receipt of Mammogram Among Mexican Migrant Women, Tijuana, Mexico, 2013^a^^, ^b

Factor	Model 1, AOR (95% CI)	Model 2, AOR (95% CI)	Model 3, AOR (95% CI)	Model 4, AOR (95% CI)
**Main predictor**				
Documented^c^ during previous 12 mo	Reference	Reference	Reference	Reference
Undocumented^d^ during previous 12 mo	0.64 (0.30–1.39)	1.01 (0.43–2.34)	1.30 (0.47–3.58)	1.14 (0.40–3.22)
**Sociodemographic characteristics**				
Age		1.04 (1.01–1.06)	1.05 (1.02–1.08)	1.06 (1.02–1.09)
Marital status				
Unmarried		Reference	Reference	Reference
Married, not living with spouse		1.54 (0.57–4.14)	2.41 (0.74–7.86)	2.75 (0.84–9.02)
Married, living with spouse		1.27 (0.68–2.37)	1.13 (0.53–2.41)	1.25 (0.57–2.72)
Completed high school		1.95 (1.02–3.75)	1.78 (0.82–3.85)	1.45 (0.65–3.23)
**Migration characteristics**				
Time spent in the United States in the previous 12 months				
30 d to <6 mo			Reference	Reference
6 mo to <12 mo			0.30 (0.09–1.03)	0.33 (0.09–1.14)
12 mo			0.45 (0.13–1.60)	0.50 (0.14–1.80)
Time in the United States during lifetime, y				
<1			Reference	Reference
1–4			0.64 (0.09–4.56)	0.68 (0.09–4.89)
5–9			0.46 (0.06–3.60)	0.45 (0.06–3.59)
≥10			1.00 (0.16–6.20)	0.70 (0.11–4.55)
**Health care access**				
Had any health insurance in previous 12 mo			4.24 (1.85–9.69)	4.17 (1.80–9.65)
Has a regular source of health care			2.62 (1.01–6.91)	2.83 (1.05–7.65)
**Acculturation**				
Level of acculturation based on language^e^				1.25 (1.01–1.55)

Abbreviation: AOR, adjusted odds ratio; CI, confidence interval.

a Data source: Project *Migrante*, which comprised a series of cross-sectional probability surveys of Mexican migrants in Tijuana from 2007 to 2015 (http://migrante.weebly.com).

b Sample restricted to migrants who had spent ≥30 days in the United States during the previous 12 months.

c Undocumented defined as all women from the deported flow and women from the southbound flow who answered positively to the question “During the last 12 months in the United States, were you undocumented any of the time?” The deported flow comprised Mexico-born Latinas returning to Mexico from the United States via deportation, and the southbound flow comprised Mexico-born Latinas recently arrived from the United States on their way voluntarily (not via deportation) to their communities of origin in Mexico.

d Documented defined as women from the southbound flow who answered no to the question “During the last 12 months in the United States, were you undocumented any of the time?”

e On a scale of 0 to 8, with 0 = lowest level of acculturation, 8 = highest level of acculturation. Scale adapted from Finch et al (15).

## Discussion

Our findings on the prevalence of cancer screening receipt support and contribute to research demonstrating that Latina migrants have lower screening rates compared with other populations. Of undocumented migrants, 22.2% reported Pap test receipt in the previous year, compared with 46.9% of documented migrants. By comparison, in the United States in 2010, the percentage of women aged 21 years or older who received a Pap test within the previous 3 years was 79.1% among non-Hispanic white women and 74.7% among Hispanic women (16) and the percentage of women aged 18 to 29 who received a Pap test within the previous year was 73.1% (17). In our study, 30.6% of undocumented migrants and 38.4% of documented migrants received a mammogram. By comparison, in 2010 in the United States, the percentage of women aged 40 or older who received a mammogram within the previous year was 51.5% among non-Hispanic white women and 46.5% among Hispanic women (16).

We found a significantly greater percentage of Pap test receipt among documented migrants than among undocumented migrants, even after adjustment for other factors. This finding is consistent with research suggesting that undocumented Latinos underuse cancer screening services (6) and expands previous research. However, we did not find a significant difference in the rate of mammogram receipt or in the likelihood of mammogram receipt between the documented and the undocumented migrant groups. Although the reason for the difference in findings between the 2 types of screenings is unclear, the findings may indicate distinct differences in the contextual factors influencing migrant women’s use of screening services, such as pregnancy status and prenatal care, different costs of the screening procedures, perception of discomfort, and level of invasiveness associated with screening procedures (18,19). Perhaps these factors play a more important role for breast cancer screening than for cervical cancer screening, rendering the role of documentation status less important in predicting the likelihood of mammography receipt.

Having a usual source of care and having health insurance are important predictors of breast and cervical cancer screening receipt (20), and undocumented status is associated with being less likely than other Latinos or whites to have medical insurance (6,14). Accordingly, in our study, undocumented migrants were less likely (but not significantly less likely) to have health insurance than were documented migrants. Mammography screening adherence is associated with having health insurance, an annual physical examination, or a recent physician visit (16,18). Insurance coverage and visits to a primary care provider within the previous year are also associated with Pap test receipt (18,21,22), whereas low rates of cervical cancer screening among immigrant women are associated with lack of a usual source of health care (23). Interestingly, in our study, having health insurance and a regular source of health care were significant predictors of mammogram receipt but not of Pap test receipt.

Latinos with high levels of acculturation use more health care services than Latinos with low levels of acculturation (13,24,25). After controlling for several factors, our study found that increased acculturation significantly predicted receipt of a mammogram but not of a Pap test. Other research suggests that higher levels of acculturation predict greater use of cervical cancer screening (26). This discrepancy might be attributed to our small sample of undocumented migrant women or to differences between circular migrants and established immigrant populations in the United States. Our sample had low levels of acculturation, but another study of Latinas in the Midwest who had low levels of acculturation showed that having a usual source of health care was a significant predictor of both mammogram and Pap test receipt (13).

That health care access and level of acculturation did not predict receipt of a Pap test might be due to several factors. We had a small sample of undocumented migrants, and we expect that a larger study would produce results consistent with other results published in the literature. The percentage of migrants who had a usual source of health care was high in this sample, considering the low levels of health insurance coverage. Furthermore, Pap test receipt is a less expensive type of screening that may be easier to schedule and complete than mammography is, making the Pap test potentially easier to obtain through community clinics, reproductive and sexual health clinics, or during other health clinic visits.

This study had several limitations. The study design may overrepresent the number of highly mobile migrants. However, circular Mexican migrants are a difficult-to-reach, understudied population, and our methods shed light on their patterns of health care use. The survey was conducted in Tijuana, and results may not apply to migrants traveling through other Mexican border regions. The response rate was moderate, and the size of the subsample of women in the survey was small, which resulted in lower than ideal sample sizes. Additional research with larger samples of migrant women is needed to estimate rates of cancer screening more accurately. Self-report of breast and cervical cancer screening may be unreliable. Future research could consider validating self-reported screening receipt with clinical records where possible. Considering the low levels of acculturation in the sample, our results on associations with acculturation level may not extrapolate to more acculturated Latina populations. Our estimates reflect and compare screening rates during the 12 months before the *Migrante* survey in 2013. Although this uniform timeframe allowed us to compare the rates of two groups, we cannot directly compare our data with data on screening rates measured during the past several years.

Despite these limitations, this study provides unique and critical data on cervical and breast cancer screening use among an understudied population of Mexican migrant women. Documented and undocumented migrant women differ in the way they use cancer screening services, and public health programs in the United States should be developed to improve rates of cancer screening among Latina migrant women, especially undocumented circular migrants. To intervene appropriately, additional research is needed to better understand the relationship between documentation status and cancer screening receipt among migrant women. Future research should aim to better understand the factors that predict cancer screening, identify potential targets for intervention to increase rates of screening receipt, and examine how the implementation of the Affordable Care Act has affected receipt of women’s preventive health care services in this population.
